# GaitRec-VR: 3D Gait Analysis for Walking Overground with and without a Head-Mounted-Display in Virtual Reality

**DOI:** 10.1038/s41597-024-03939-0

**Published:** 2024-10-08

**Authors:** Mark Simonlehner, Bernhard Dumphart, Brian Horsak

**Affiliations:** 1 Center for Digital Health & Social Innovation, St. Pölten University of Applied Sciences, St. Pölten, Austria; 2 Institute of Health Sciences, Department of Health, St. Pölten University of Applied Sciences, St. Pölten, Austria

**Keywords:** Health care, Public health

## Abstract

This data descriptor introduces *GaitRec-VR*, a 3D gait analysis dataset consisting of 20 healthy participants (9 males, 11 females, age range 21–56) walking at self-selected speeds in a real-world laboratory and the virtual reality (VR) replicas of this laboratory. Utilizing a head-mounted display and a 12-camera motion capture system alongside a synchronized force plate, the dataset encapsulates real and virtual walking experiences. A direct kinematic model and an inverse dynamic approach were employed for kinematics and computation of joint moments respectively, with an average of 23 ± 6 steps for kinematics and five clean force plate strikes per participant for kinetic analysis. *GaitRec-VR* facilitates a deeper understanding of human movement in virtual environments, particularly focusing on dynamic balance during walking in healthy adults, crucial for effective VR applications in clinical settings. The dataset, available in both.c3d and.csv formats, allows further exploration into VR’s impact on gait, bridging the gap between physical and virtual locomotion.

## Background & Summary

Augmented, mixed, and virtual reality (VR) technologies have revolutionized the way we perceive and interact with our environment. The global market for these technologies has witnessed a meteoric rise, with projections estimating its worth to be nearly €800 billion by 2030, with the healthcare sector being a significant beneficiary of this growth^1^. VR’s potential in gait training and rehabilitation is particularly noteworthy. This technology offers an immersive experience, enhancing motivation, and targeting specific gait parameters, making it a valuable addition to traditional therapy approaches^2,3^.

3D motion analysis is a technique that captures and evaluates the three-dimensional movements of the human body. It provides insights into the biomechanics of movements, such as walking, and is crucial for understanding gait patterns and abnormalities. Conversely, VR is a simulated experience that can either mirror the real world or diverge from it entirely. The integration of 3D motion analysis with VR has opened up new possibilities for understanding human movement in virtual environments. However, the combined potential of VR and 3DGA is not yet adequately researched, presenting a gap in our knowledge. This lack of comprehensive research is an issue we want to address with this dataset^4^.

Patients with gait and balance disorders, such as Parkinson’s Disease^5^, Cerebral Palsy^6^, and stroke^7^, have benefited from VR interventions. Most VR applications in gait rehabilitation have been on treadmills with semi-immersive setups. However, the advent of head-mounted displays (HMD) has opened up new possibilities, allowing patients to walk overground in virtual environments, offering a more realistic and unrestricted experience^8^.

Yet, before fully immersive overground VR environments can be deemed effective and safe for populations with gait and balance disorders, it is essential to understand how VR impacts dynamic balance during walking in healthy adults. Dynamic balance is the ability to maintain stability while in motion, a critical aspect of walking. The neuromuscular system plays a pivotal role in ensuring dynamic balance, counteracting gravitational and joint reaction forces to prevent falls^9,10^.

The use of HMDs in VR can alter the visual input, potentially leading to sensory conflicts. This discrepancy between virtual visual input and real-world sensory cues, such as vestibular and proprioceptive feedback, can influence stable walking^11^. Given the significance of these effects, understanding VR’s impact on gait is vital for its therapeutic and rehabilitative applications. To delve deeper into this, we developed the *GaitRec-VR* dataset^4^ to further investigate VR’s impact on gait, particularly in the context of biomechanics. This dataset, available in.c3d and.csv formats, encapsulates data from four distinct experimental conditions, facilitating a comprehensive analysis of human gait patterns in various simulated environments and offering a comparison between real and replicated laboratory settings.

Our research has previously explored the biomechanical dynamics of gait within variously sized virtual reality (VR) environments and the fidelity of replicated laboratory conditions^12,13^. Initial findings indicated a cautious gait strategy in VR settings, potentially attributed to the perceived instability of virtual environments^12^. Further investigation highlighted modifications in trunk kinematic variability and dynamic balance during VR-assisted walking, suggesting adjustments in center of mass and foot placement to enhance stability^13^.

With the release of the revised *GaitRec-VR* dataset^4^, our aim is to enable research into the effects of real and simulated environmental dimensions on overground gait biomechanics, providing detailed comparisons between actual laboratories and their VR replicas. This dataset offers insights into the biomechanical consequences of environmental scaling. This effort is further enhanced by utilizing *IntellEvent*^14^, a novel algorithm based on velocity data for the improved detection of gait events. Such enhancement is crucial for the accurate biomechanical analysis of walking patterns within the domains of biomechanics and virtual reality research. This manuscript not only builds upon our previous work but also includes a comprehensive review of the dataset, a description of all available data, and updates to methodologies, thereby increasing the dataset’s precision and expanding its applicability in research. Through these enhancements, the *GaitRec-VR* dataset^4^ emerges as a more robust resource, promising to contribute to the fields of biomechanics and immersive virtual reality. By improving the accuracy of gait event detection and broadening the analytical scope, it enables richer and more insightful investigations into human gait dynamics in both physical and virtual settings.

## Methods

### Participants

A total of 20 healthy volunteers, comprising 9 males and 11 females, were conveniently selected from our university’s campus. These participants were the same as those in our previous studies^12,13^. They had an average age of 37.2 ± 8.7 years, weight of 73.9 ± 15.7 kg, and height of 170.3 ± 6.6 cm. the values for each participant can be seen in Table 1. Eligibility criteria included an age range of 21 to 56 years. Those with any temporary or chronic conditions that could impair walking were not considered. To ensure adherence to the exclusion criteria, a physiotherapist conducted a thorough checkup prior to the measurements. The research was executed with the endorsement of the local ethics committee (GS1-EK-4/682-2020) and in compliance with pertinent guidelines and regulations. All participants provided informed consent before joining the study.

**Table 1:** This table presents the individual anthropometric data of the 20 healthy volunteers (9 males and 11 females) who participated in the study.

Name	Body Mass [kg]	Height [cm]	Age	Sex
N002	57.5	169	44	female
N003	66.5	167	24	female
N004	108.7	176	42	male
N005	62.8	167	47	female
N007	61	167	29	male
N008	71.8	174	42	male
N009	100.5	182	38	male
N010	60.7	165	42	female
N011	71	167	24	female
N015	67.5	162	37	female
N016	94	186	41	male
N018	54	166	32	female
N019	67.9	166	56	female
N020	98	178	38	male
N021	61.9	158	21	female
N022	64.7	170	35	male
N023	85.5	172	46	male
N024	67	169	34	female
N025	74.6	173	41	male
N026	82.1	172	30	female

The participants, who were selected from our university’s campus, had an average age of 37.15 ± 8.72 years, a weight of 73.89 ± 15.65 kg, and a height of 170.30 ± 6.63 cm. They were within the age range of 21 to 56 years and had no conditions that could impair walking, as verified by a physiotherapist.

### Study protocol

All participants underwent a standard 3D gait analysis in four different walking conditions, with the order of these conditions randomly assigned to each participant to control for potential order effects, following the protocol established in our previous works^12,13^. These conditions included the real laboratory (RLab), a virtual laboratory resembling the real world (VRLab, 11.9 × 5.4 m), a smaller version of the VRLab (VRLab−, 8.7 × 5.4 m), and a version which was twice as long as the VRLab (VRLab+, 23.5 × 5.4 m). The first two conditions were designed to evaluate the effect of overground walking in a VR environment on selected gait variables. The last two virtual environments (VRLab + vs. VRLab−) were designed to investigate the influence of the physical dimension of the VR environment, i.e., the size of the room, on self-selected walking speed. The walkway length, positioned approximately in the center of the real and all virtual environments, was approximately 7 m long and was consistent across all four conditions.

Before data recording, participants underwent a 5-minute acclimatization period to familiarize themselves with the environment and the VR-HMD. During this period, they practiced walking in a straight line along the designated walkway, reaching the end, turning around, and walking back to the starting point. This practice walking was identical to the walking pattern used during the data capturing phase, ensuring comfort and ease of movement within the experimental setup. Additionally, they were instructed to maintain their normal comfortable walking speed throughout the experiment. Participants wore the HMD for approximately 7 ± 1 minutes per VR condition, totaling approximately 21 minutes during the entire study. This protocol ensured that the data collected was representative of the participants’ natural gait in both real and virtual environments.

During the data collection, participants continued to walk in a straight line along the walkway, reaching the end, turning around, and walking back to the starting point. Due to the constraints of the capturing volume of our motion capture system, the turn at the end of each walk was not recorded. The data analysis did not differentiate between the outbound and return phases.

### The virtual overground environment

**Fig. 1:**
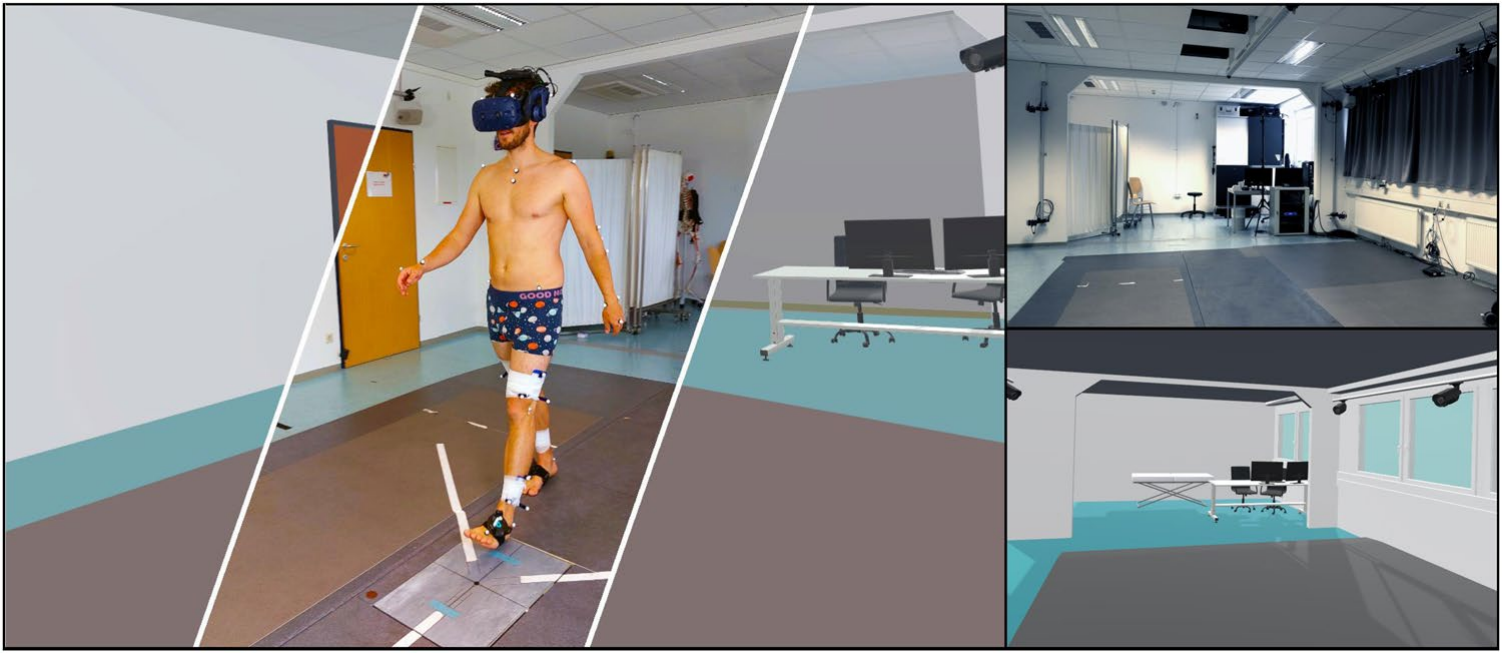
The left image showcases both the real laboratory and the virtual replica of the laboratory on either side. Positioned in the center is a participant wearing VR goggles and trackers on their feet, with spherical markers attached to their body. The participant is captured in the act of stepping onto the force plate that is being utilized. The upper right image shows the real laboratory and the lower right side the Virtual replica. Images partly reused from Horsak *et al*.^13^.

The virtual reality (VR) environment was presented to participants using a wirelessly operated head-mounted display (HMD, HTC Vive Pro) that was calibrated to the real-world (Fig. 1). HTC Vive 2.0 trackers were attached to the participants’ feet to track and display their feet movements in real-time within the VR environment. Five HTC Vive Lighthouses (2.0) were positioned in the laboratory to continuously track the positions of the HMD and the trackers. We created a virtual 3D model of our laboratory, including part of its interior, based on its real physical dimensions. The Unity3D game engine was utilized for real-time visualization of the laboratory in VR and for developing the VR application.

**Fig. 2:**
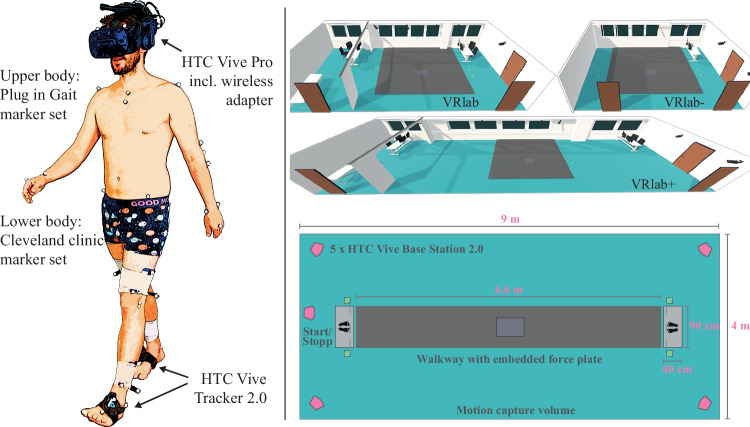
The left side of the image depicts a participant wearing VR goggles and trackers on their feet, along with optical markers attached to their body. On the right side, at the top, are virtual replicas of the laboratory. These replicas include the laboratory in the same size as the real room (VRLab) with the dimensions 11.9 × 5.4 m, a scaled-down version (VRLab−) with the dimensions 8.7 × 5.4 m, and an enlarged version (VRLab+) with the dimensions 23.5 × 5.4 m. The bottom right portion of the image illustrates the size of the walkway and the capture volume. The pink pentagons indicate the positions of the HTC Vive Base Stations. Images partly reused from Horsak *et al*.^12^.

Three different sized virtual replicas of the laboratory were created to accommodate varying experimental needs (Fig. 2). The first replica, called VRLab, had dimensions of 11.9 × 5.4 m, closely resembling the real-world laboratory. A smaller version of the VRLab, named VRLab−, was designed with dimensions of 8.7 × 5.4 m. Additionally, a larger version, named VRLab+, was developed, which was twice as long as the VRLab, measuring 23.5 × 5.4 m.

### 3D Gait analysis

**Fig. 3:**
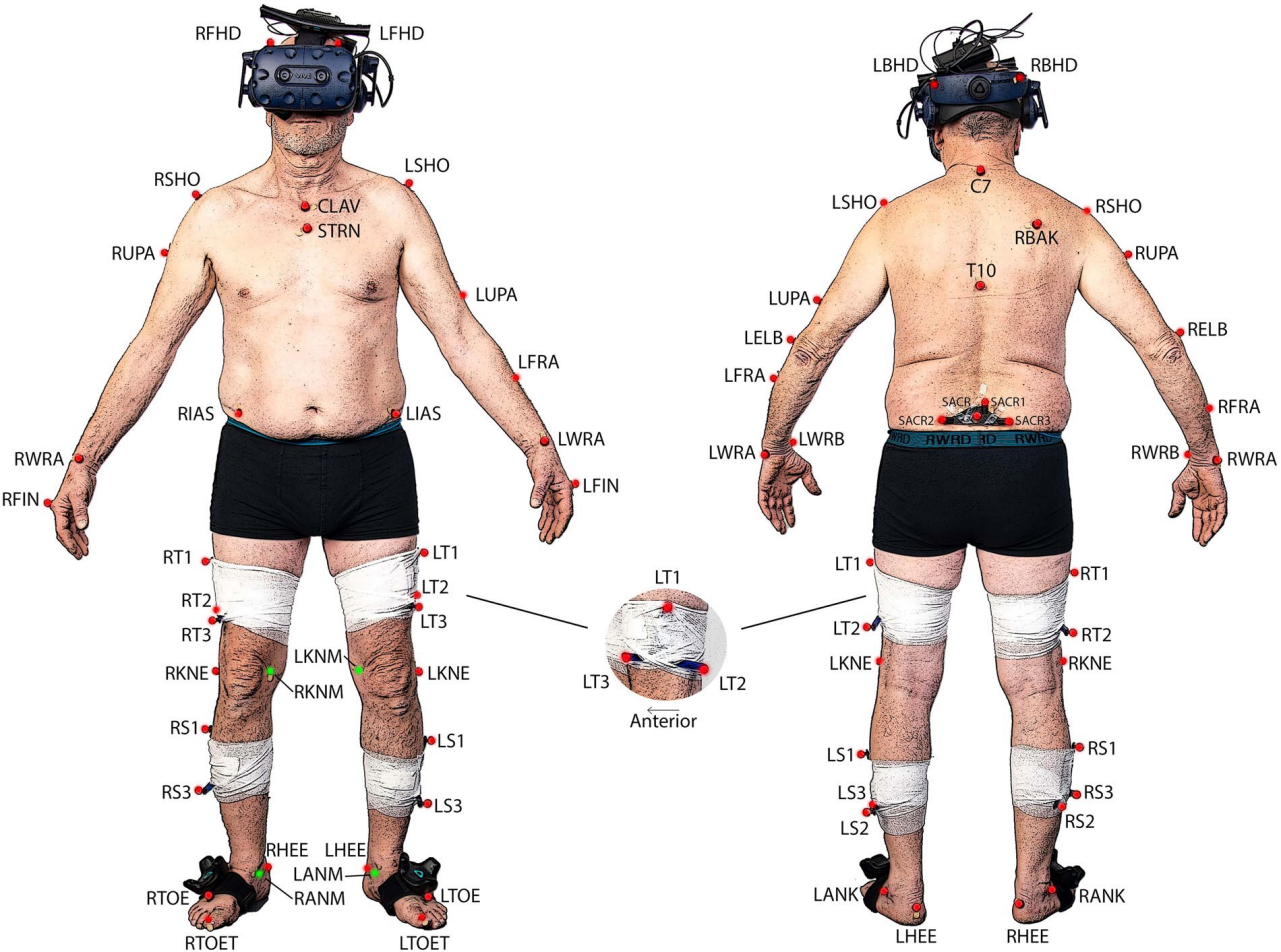
Position and names of the spherical markers that were used to record the data are illustrated in this figure. The extended Cleveland Clinic marker set was utilized for the lower extremity, combined with the Vicon Plug-In-Gait model for the upper body. The marker spheres, highlighted in green, were used solely for calibration purposes and to define the frontal axis of the lower limb. For subsequent trials, these spheres were detached. Central to the illustration is the tracking cluster that is attached to both legs at the thigh and shank, showcased as a representative example from a side view perspective.

**Fig. 4:**
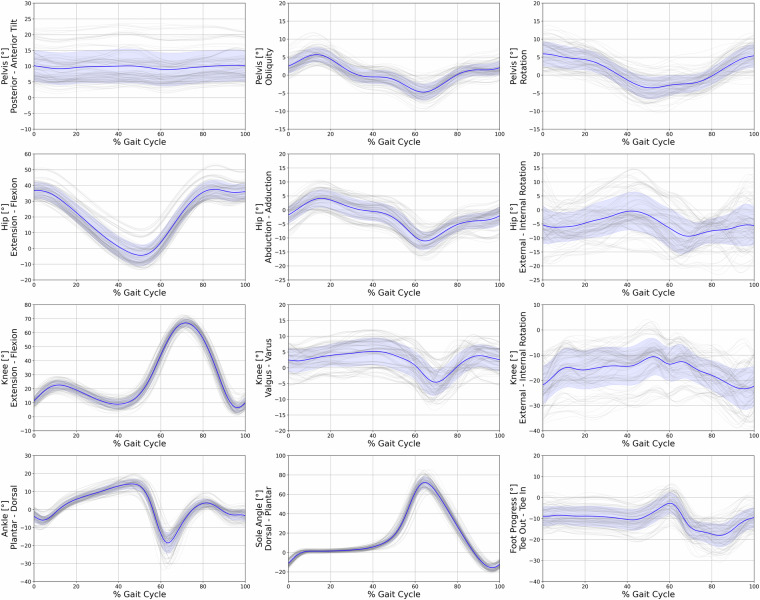
Kinematic data of lower extremity joint angles for the pelvis, hip, knee, ankle, sole, and foot progression during the gait cycle, from all trials of all participants while walking in the Real Laboratory (RLab). The x-axis represents the percentage of the gait cycle, while the y-axis indicates the joint angles in degrees. Individual walking trials are depicted in grey, with the blue line representing the mean angle of all trials, and the blue band representing the standard deviation.

**Fig. 5:**
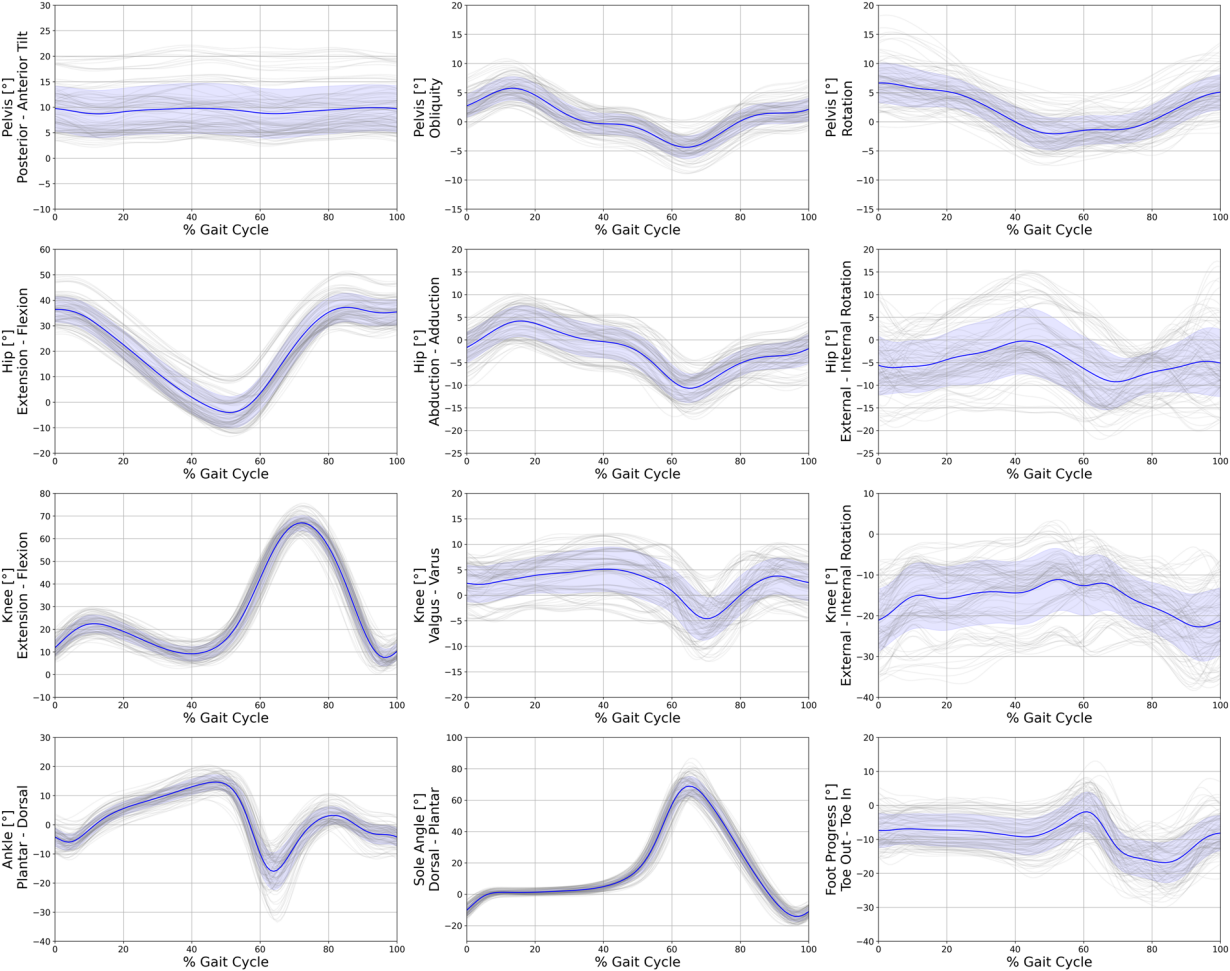
Kinematic data of lower extremity joint angles for the pelvis, hip, knee, ankle, sole, and foot progression during the gait cycle, from all trials of all participants while walking in the Virtual Laboratory (VRLab). The x-axis represents the percentage of the gait cycle, while the y-axis indicates the joint angles in degrees. Individual walking trials are depicted in grey, with the blue line representing the mean angle of all trials, and the blue band representing the standard deviation.

**Fig. 6:**
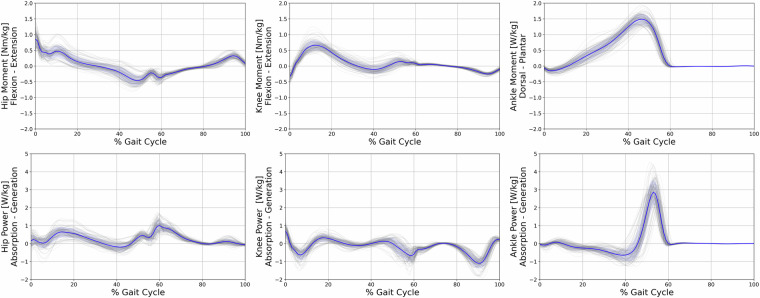
Kinetic data of lower extremity joint moments and power for the hip, knee, and ankle during the gait cycle, from trials conducted in the Real Laboratory (RLab). The x-axis represents the percentage of the gait cycle, while the y-axis indicates the joint moments in Nm/kg and power in W/kg. Individual walking trials are depicted in grey, with the blue line representing the mean moment of all trials, and the blue band representing the standard deviation.

**Fig. 7:**
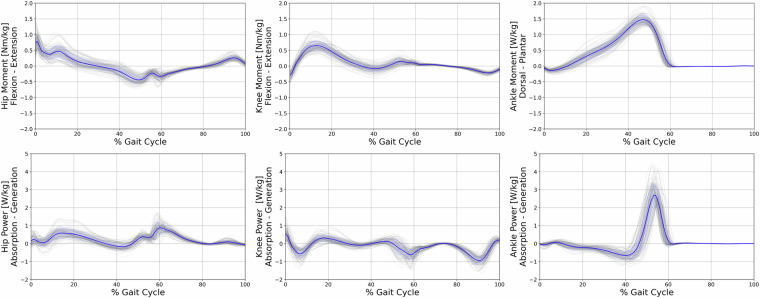
Kinetic data of lower extremity joint moments and power for the hip, knee, and ankle during the gait cycle, from trials conducted in the Virtual Laboratory (VRLab). The x-axis represents the percentage of the gait cycle, while the y-axis indicates the joint moments in Nm/kg and power in W/kg. Individual walking trials are depicted in grey, with the blue line representing the mean moment of all trials, and the blue band representing the standard deviation.

A Vicon 12-camera motion capture system using the Vicon Nexus Software (Nexus, 2.11, Vicon, Oxford, UK) was used to collect trajectory data at a sampling rate of 150 Hz. Concurrently, ground reaction force data were recorded using a synchronized force plate (Kistler, Winterthur, CH) at a sampling rate of 900 Hz. The extended Cleveland Clinic marker set was employed for lower extremity kinematics, in conjunction with the Vicon Plug-In-Gait model for the upper body^15^. The marker positions and names can be referred to in Fig. 3. To determine the hip joint center, the regression equation developed by Davis^16^ was utilized. In order to establish the frontal axis of the lower limb, lateral and medial markers were positioned on the Epicondylus of the knee and ankle. The use of these markers in gait analysis has been widely documented in previous studies, reinforcing the validity of this approach^17^. When utilizing the head-mounted display during the experiment, it was necessary to reposition the head markers directly onto the HMD. To counteract any potential bias introduced by this adjustment, we conducted a calibration trial for each experimental condition. During these trials, participants were instructed to maintain a stationary stance with their heads in a neutral position. The data from these calibration trials were then used as a reference to recalibrate the head and neck angles in the subsequent experimental trials. These trials are included in the dataset^4^, with the dynamic C3D files containing the corrected head poses. Kinetic data were filtered using a 4th order zero-lag Butterworth filter^18^ with a cut-off frequency of 20 Hz. Raw kinematic trajectories were filtered using the Woltring filtering routine^19^ integrated into the Vicon Nexus system, with a mean squared error (MSE) value of 15. In total, five clean force plate strikes per body side were recorded for each condition. For kinematic variables, however, all available steps before and after the force plate were used, resulting in an average of 23 ± 6 steps per participant, condition, and side. Figures 4, 6 present the file content with kinematic and kinetic data from RLab trials, while Figs. 5, 7 display the corresponding data from VRLab trials.

### Event detection

In the revised *GaitRec-VR* dataset^4^, we have implemented improvements to enhance both the dataset’s accuracy in event detection and its overall comprehensiveness. These enhancements are built upon the foundational data outlined in citations^12^ and^13^. A key advancement in our methodological approach is the incorporation of *IntellEvent*^14^, a novel, deep learning-based algorithm designed for the precise identification of initial contact (IC) and foot off (FO) events. This algorithm conducts a analysis of the velocity data from the left and right ankle, heel, and toe markers to accurately determine these gait events. Following the algorithm’s automated event identification, the accuracy of these events was verified and manually corrected as necessary by two expert assessors, ensuring the highest level of precision in our gait event detection process.

## Data Records

The dataset can be downloaded at PHAIDRA^4^. The data is organized into individual folders for each recorded participant. Each folder is named using the format N0XX, where ‘N’ stands for number and ‘XX’ denotes a randomly assigned ID encompassing all the evaluated participants. The data can be accessed in both .c3d and .csv file formats.The folders are organized into three categories: “Stand”, “Static”, and “Dynamic”, each containing distinct types of files:

- **Stand Files**: These contain recordings of participants assuming an “A” pose on the force plate. This pose is characterized by standing with feet hip-width apart and hands slightly extended to the sides, as illustrated in Fig. 3. Before each session, calibration trials are conducted, labeled as “ZSX”, where ‘X’ corresponds to the subsequent testing condition. For example, “ZSA” would denote a calibration trial conducted before testing in the actual laboratory setting.
- **Static Files**: Similar to the “Stand” files, these record the “A” pose but with a modification of slightly bent knees.
- **Dynamic Files**: These encompass the recordings of gait sessions.

Each file name includes a letter and a sequential number, which indicate the experiment’s condition and the progression of the session, respectively. The letters signify the following:

- **A**: The actual laboratory setting.
- **B**: The VRLab, a virtual replica of the laboratory with dimensions of 11.9 × 5.4 m, closely resembling the real-world laboratory.
- **C**: The VRLab−, a smaller version of the VRLab with dimensions of 8.7 × 5.4 m.
- **D**: The VRLab+, a larger version which is twice as long as the VRLab, measuring 23.5 × 5.4 m.

### C3D file content

The C3D file contains the following data:

- **nFrame**: Number of frames in the region of interest.
- **iFrame**: First frame of the region of interest.
- **fFrame**: Last frame of the region of interest.
- **VideoFreq**: Frequency of trajectory capturing.
- **AnalogRate**: Number of Ground Reaction Force (GRF) subframes.
- **AnalogFreq**: Frequency of GRF data capturing.
- **Traj**: Trajectories of every physical marker sphere and modeled trajectories. The label data field gives the position of the corresponding name in the value data field with the format Frame x Label x Three-Dimensional Position Data in mm.
- **FP**: Ground reaction forces with the force in the data field **F** represented as Frame x Three-Dimensional Force Data in N, and the center of pressure in the data field **CoP** represented as Frame x Three-Dimensional Position Data in mm.
- **Event**: Heel strike (HS) and toe off (TO) event data. FrameOffset defines the frame the frame number in **RTO,** **LTO,** **RHS**, and **LHS** to which it is referred. **R** stands for the right foot, and **L** for the left foot.
- **Processing**: Contains parameters describing the participant. In addition to values required for internal calculations, general data such as body mass, height, and various length and width measurements of the participant are included.
- **TDP,** **EMG**, and **Aux**: The variables presented are placeholders, as this specific data was not acquired during our experimental trials.
- **Model**: Randomly assigned ID for each recorded participant.

The provided names are merely illustrative, meant to represent the content within the file. The specific labels for individual data fields might differ based on the software utilized to access the file. Nevertheless, given that C3D is a standardized format, you have the flexibility to import and process the data using your software of choice.

### Content of the CSV file

The CSV file contains the following data:

- **Events**: The first row after the event header shows the frequency of trajectory capturing. The following rows and columns contain the Heel strike (HS) and Toe off (TO) event data in seconds.
- **Devices**: The first row after the device header shows the frequency of Ground Reaction Force (GRF) capturing. The following rows and columns contain the corresponding frame number, force, moment, and center of pressure (CoP) as three-dimensional data, including units.
- **Model Outputs**: The first row after the event header shows the frequency of trajectory capturing. The following rows and columns contain values required for internal calculations and internal forces, moments, angles, and powers as three- or six-dimensional data, including units.
- **Trajectories**: The first row after the event header shows the frequency of trajectory capturing. The following rows and columns contain trajectories of every physical marker sphere as three-dimensional data, including units.

## Technical Validation

To ensure consistency in the marker data, the placement of marker spheres on the patients was performed exclusively by the first two authors and cross-validated by each other. The hardware configuration remained consistent across all recordings, and the system underwent calibration before each measurement session. Any trajectory gaps resulting from masking were addressed by the first two authors using spline and pattern fill techniques within Vicon Nexus software.

For synchronization purposes, the force plate was seamlessly integrated into Vicon Nexus using a native SDK provided by Kistler. This integration allowed for accurate alignment of the force plate recordings with the marker system data.

## Usage Notes

The C3d format is a widely used standard in the fields of biomechanics, animation, and gait analysis. Detailed documentation for this format can be found on the website www.c3d.org. To open and work with C3d files, open-source freeware tools such as Motion Kinematic and Kinetic Analyzer (MOKKA)^20^ (available at https://biomechanical-toolkit.github.io/mokka/) or EZC3D^21^ can be utilized.

## Data Availability

The code for event calculation is found at https://github.com/fhstp/IntellEvent. For parsing c3d files, you have the option of using EZC3D (https://github.com/pyomeca/ezc3d) and the Biomechanics Toolkit (BTK) (https://github.com/Biomechanical-ToolKit/BTKCore), both of which are freely available.

## References

[1] Commission, E. Virtual worlds and web 4.0 - factsheet. https://digital-strategy.ec.europa.eu/en/library/virtual-worlds-and-web-40-factsheet. Accessed: 2023-07-25 (2023).

[2] Canning, C. G. *et al*. Virtual reality in research and rehabilitation of gait and balance in Parkinson disease. *Nature Reviews Neurology* **16**, 409–425, 10.1038/s41582-020-0370-2 (2020). 32591756 10.1038/s41582-020-0370-2

[3] Janeh, O. & Steinicke, F. A review of the potential of virtual walking techniques for gait rehabilitation. *Frontiers in Human Neuroscience* **15** (2021). 10.3389/fnhum.2021.717291 PMC8595292 34803632

[4] Simonlehner, M., Dumphart, B. & Horsak, B. GaitRec-VR: 3D Gait Analysis for Walking Overground with and without a Head-Mounted-Display in Virtual Reality. *Phaidra*10.60522/o:5587 (2024). 10.1038/s41597-024-03939-0 PMC11461699 39379382

[5] Triegaardt, J. *et al*. The role of virtual reality on outcomes in rehabilitation of parkinson’s disease. *Neurological Sciences* **41**, 529–536 (2020). 31808000 10.1007/s10072-019-04144-3 PMC7040061

[6] de Oliveira, J. M. *et al*. Novel virtual environment for alternative treatment of children with cerebral palsy. *Computational intelligence and neuroscience* **2016** (2016). 10.1155/2016/8984379 PMC4923569 27403154

[7] Palacios-Navarro, G. & Hogan, N. Head-mounted display-based therapies for adults post-stroke. *Sensors* **21** (2021). 10.3390/s21041111 PMC7915338 33562657

[8] Ghai, S., Ghai, I. & Lamontagne, A. Virtual reality training enhances gait poststroke: a systematic review and meta-analysis. *Annals of the New York Academy of Sciences* **1478**, 10.1111/nyas.14420 (2020). 10.1111/nyas.14420 32659041

[9] Winter, D. Human balance and posture control during standing and walking. *Gait & Posture* **3**, 193–214, 10.1016/0966-6362(96)82849-9 (1995).

[10] MacKinnon, C. & Winter, D. Control of whole body balance in the frontal plane during human walking. *Journal of Biomechanics* **26**, 633–644, 10.1016/0021-9290(93)90027-C (1993). 8514809 10.1016/0021-9290(93)90027-c

[11] Palmisano, S. *et al*. Differences in virtual and physical head orientation predict sickness during active head-mounted display-based virtual reality. *Virtual Reality* **27**, 1293–1313, 10.1007/s10055-022-00732-5 (2023). 36567954 10.1007/s10055-022-00732-5 PMC9761034

[12] Horsak, B. *et al*. Overground Walking in a Fully Immersive Virtual Reality: A Comprehensive Study on the Effects on Full-Body Walking Biomechanics. *Frontiers in Bioengineering and Biotechnology* **9**, 10 (2021). 10.3389/fbioe.2021.780314 PMC8693458 34957075

[13] Horsak, B., Simonlehner, M., Dumphart, B. & Siragy, T. Overground walking while using a virtual reality head mounted display increases variability in trunk kinematics and reduces dynamic balance in young adults. *Virtual Reality*10.1007/s10055-023-00851-7 (2023).

[14] Dumphart, B. *et al*. Robust deep learning-based gait event detection across various pathologies. *PLOS ONE* **18**, e0288555, 10.1371/journal.pone.0288555 (2023). 37566568 10.1371/journal.pone.0288555 PMC10420363

[15] Svoboda, B. & Kranzl, A. A study of the reproducibility of the marker application of the cleveland clinic marker set including the plug-in gait upper body model in clinical gait analysis. *Gait & Posture* **35**, 535–540, 10.1016/j.gaitpost.2011.10.286 (2012).22197290

[16] Davis, R. B. III, Ounpuu, S., Tyburski, D. & Gage, J. R. A gait analysis data collection and reduction technique. *Human movement science* **10**, 575–587 (1991). Publisher: Elsevier.

[17] Kadaba, M. P., Ramakrishnan, H. K. & Wootten, M. E. Measurement of lower extremity kinematics during level walking. *Journal of Orthopaedic Research* **8**, 383–392 (1990). 2324857 10.1002/jor.1100080310

[18] Butterworth, S. On the theory of filter amplifiers. *Wireless Engineer* **7**, 536–541 (1930).

[19] Woltring, H. A fortran package for generalized, cross-validatory spline smoothing and differentiation. *Advances in Engineering Software* **8**, 104–113 (1986).

[20] Barré, A. Mokka - Motion Kinematic & Kinetic Analyzer (2013).

[21] Michaud, B. & Begon, M. ezc3d: An easy C3D file I/O cross-platform solution for C++, Python and MATLAB. *Journal of Open Source Software* **6**, 2911, 10.21105/joss.02911 (2021).

